# Scientists’ sense making when hypothesizing about disease mechanisms from expression data and their needs for visualization support

**DOI:** 10.1186/1471-2105-15-117

**Published:** 2014-04-26

**Authors:** Barbara Mirel, Carsten Görg

**Affiliations:** 1 School of Education, University of Michigan, Ann Arbor, Michigan 48109, USA; 2 Computational Bioscience Program, School of Medicine, University of Colorado, Denver, Colorado, USA

**Keywords:** Sense making, Usability, Complex workflow, Cognitive tasks, Systems biology, Network analysis, Visualization, Qualitative research, Case study

## Abstract

A common class of biomedical analysis is to explore expression data from high throughput experiments for the purpose of uncovering functional relationships that can lead to a hypothesis about mechanisms of a disease. We call this analysis expression driven, -omics hypothesizing. In it, scientists use interactive data visualizations and read deeply in the research literature. Little is known, however, about the actual flow of reasoning and behaviors (sense making) that scientists enact in this analysis, end-to-end. Understanding this flow is important because if bioinformatics tools are to be truly useful they must support it. Sense making models of visual analytics in other domains have been developed and used to inform the design of useful and usable tools. We believe they would be helpful in bioinformatics. To characterize the sense making involved in expression-driven, -omics hypothesizing, we conducted an in-depth observational study of one scientist as she engaged in this analysis over six months. From findings, we abstracted a preliminary sense making model. Here we describe its stages and suggest guidelines for developing visualization tools that we derived from this case. A single case cannot be generalized. But we offer our findings, sense making model and case-based tool guidelines as a first step toward increasing interest and further research in the bioinformatics field on scientists’ analytical workflows and their implications for tool design.

## Background

A common class of exploratory analysis is to examine the functions of differentially expressed genes from high throughput experiments using interactive protein-protein interactions networks, visual pathways, and other bioinformatics applications. The goal is to generate a hypothesis about molecular mechanisms of a disease. We call this analysis expression-driven, -omics hypothesizing. Despite the availability of many interactive data visualizations to support the graphic portions of this analysis most of the tools do not adequately address scientists’ actual analytical needs and practices. They need to be more useful and usable and better integrated [1].

In other domains, improvements in data visualization designs have relied on models of analysts’ actual sense making for a complex analysis [2]. A sense making model captures analysts’ cumulative, looped (not linear) “process [es] of searching for a representation and encoding data in that representation to answer task-specific questions” relevant to an open-ended problem [3]: 269. As an end-to-end flow of application-level tasks, a sense making model may portray and categorize analytical intentions, associated tasks, corresponding moves and strategies, informational inputs and outputs, and progression and iteration over time. The importance of sense making models is twofold: (1) If an analytical problem is poorly understood developers are likely to design for the wrong questions, and tool utility suffers; and (2) if developers do not have a holistic understanding of the entire analytical process, developed tools may be useful for one specific part of the process but will not integrate effectively in the overall workflow [4,5].

In the visual analytics research literature Pirolli and Card’s sense making model of intelligence analysis is a seminal model that has led to positive tool designs and has inspired other models in different domains [6-12]. Unfortunately, little is empirically known or modelled in biomedicine about the end-to-end flow of expression-driven, -omics hypothesizing. Some research describes programmatic operations for just a portion of it, for example, describing the processes of identifying protein complexes as clustering networks, sub-dividing by attributes, assigning color or shape to functional dimensions, and creating biologically meaningful layouts [1]. But researchers have not empirically described the integrated thinking and behaviors that occur within and across the stages of the whole analysis. These stages include visual analytics as well as deep reading in the research literature.

Our goal is to empirically capture the end-to-end sense making flow of expression-driven, -omics hypothesizing. Working towards this goal is a continuous project. Here we report on a preliminary model that we derived from an initial field-based observational study. Observational research is an appropriate first step when knowledge is slight - as it is with this workflow [13]. The benefit of a field study is that it can uncover and qualitatively describe the flow and categories of reasoning and behaviors characterizing complex explorations. It also can reveal gaps in tool designs that impede researchers’ cumulative analysis. Such results can establish a necessary foundation on which to incrementally build and validate a generalizable sense making model, which, in turn, can inform the evaluation of tools.

We studied in-depth one biomedical researcher as she engaged in multiple stages of expression-driven, -omics hypothesizing over a six month span. To our knowledge, our results are the first end-to-end description of sense making for this class of analysis – that is, its actual looped analytical processes and intentions; behaviors and reasoning with visualizations; engagement with numerous tools (visual and textual); research into the literature coupled with visual analytics; and various modes and content of note-taking. We recognize that this one case cannot be generalized. Yet we believe that it is a first step and can inform further research toward this end. Moreover, to lend reliability to our findings we tied them whenever possible to relevant results from our own prior research and from studies by others [14-18]. This commentary reports on this synthesis. It presents the resulting sense making model, describes it in detail, and recommends guidelines that we derived from our one case for tool design.

### A sense making model for hypothesizing about disease mechanisms: an overview

**Figure 1:**
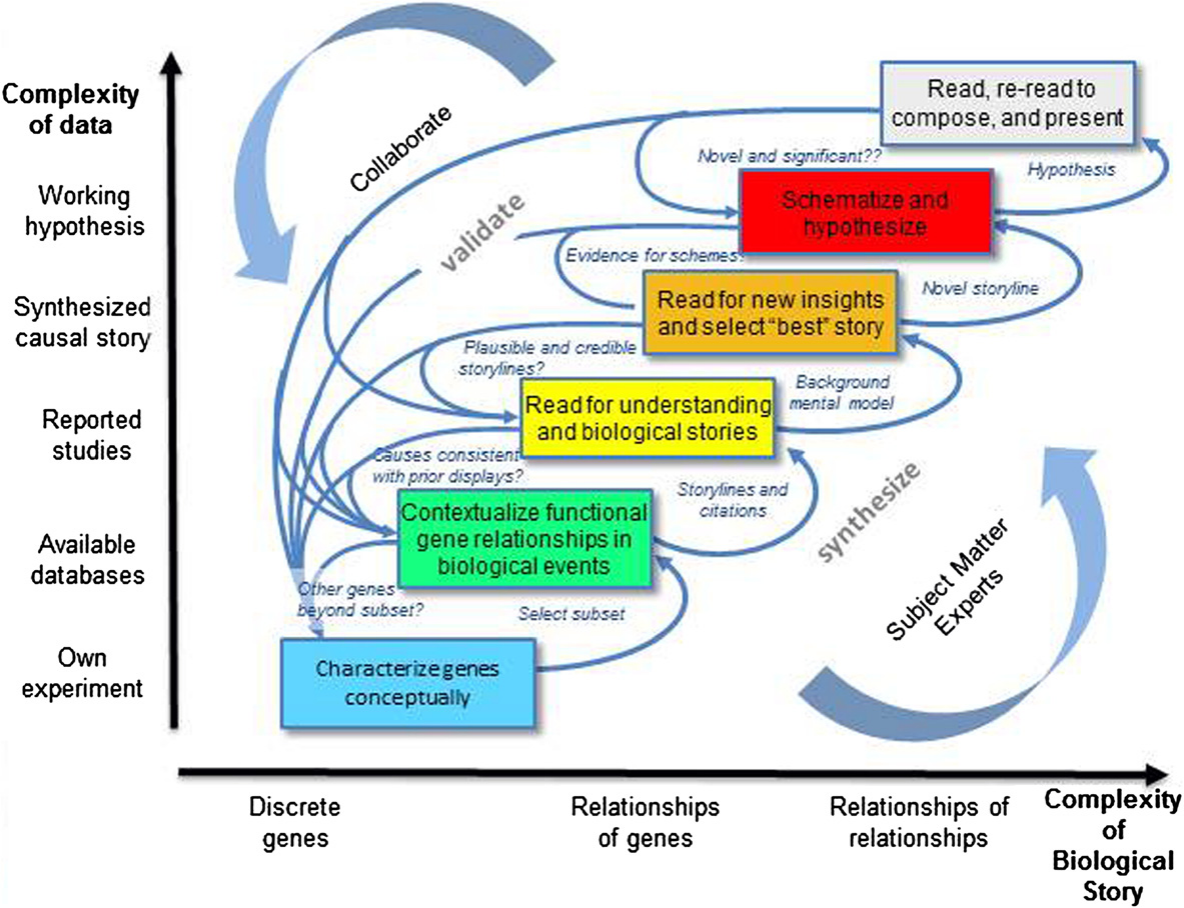
**Sense making model for exploring molecular level influences on disease-related processes.** Our model presents analysts’ progressive and cumulative sources of data for constructing and representing knowledge on the y-axis and their progressive units (or focus) of analysis on the x-axis. It names stages by users’ analysis objectives not by information artifacts as in the Pirolli and Card model; and it ties stages/objectives to domain content. It also includes validation questions analysts iteratively investigate (arrow chains from higher to lower stages); and output from each stage that serves as input for the next.

We abstracted six stages of end-to-end sense making and roughly quantified the duration of each based on the case study researcher’s demonstrated and reported engagement. To represent these stages visually, we adapted Pirolli and Card’s graphic representation of a sense making model expression-driven, -omics hypothesizing (see Figure 1).

In order to center our model on the user, we structured its stages by researchers’ analytical intentions, unlike Pirolli and Card’s focus on information per se. In later sections we discuss each stage separately accompanied by figures that capture activities in each stage. For these figures we adapted Brehmer and Munzer’s (2013) graphic framework for capturing and naming visual analytic tasks [19]. This framing takes a user-centered perspective by highlighting scientists’ purposes for interacting with visualizations – the “why” of a class of analysis – and tying them to “how” scientists interact and with the tools (software-supported methods) and “what” data and objects are involved. The names we use for the “why” and “how” activities are informed, as well, by Yi et al’s categories of visual analytic tasks [20]. These systems for classifying tasks from both Brehmer and Munzer and Yi et al. trace to years of extensive research in visual analytics that aims to define users’ work in ways that can inform tool design.

As a caveat, neither our prose descriptions nor figures of stages can fully capture the iterative and opportunistic analysis that actually occurs within and across stages. Nor do they do justice to researchers’ interleaving of metacognitive processes with logical analysis. Metacognitive processes involve efforts to manage evolving knowledge; monitor progress; and validate sources, data, and interpretations. For a narrative retelling of the exploratory analysis and scientific context see Additional file 1: Heart Failure Case Study: Narrative of Progressive Discovery.

### Duration of sense making stages

**Figure 2:**
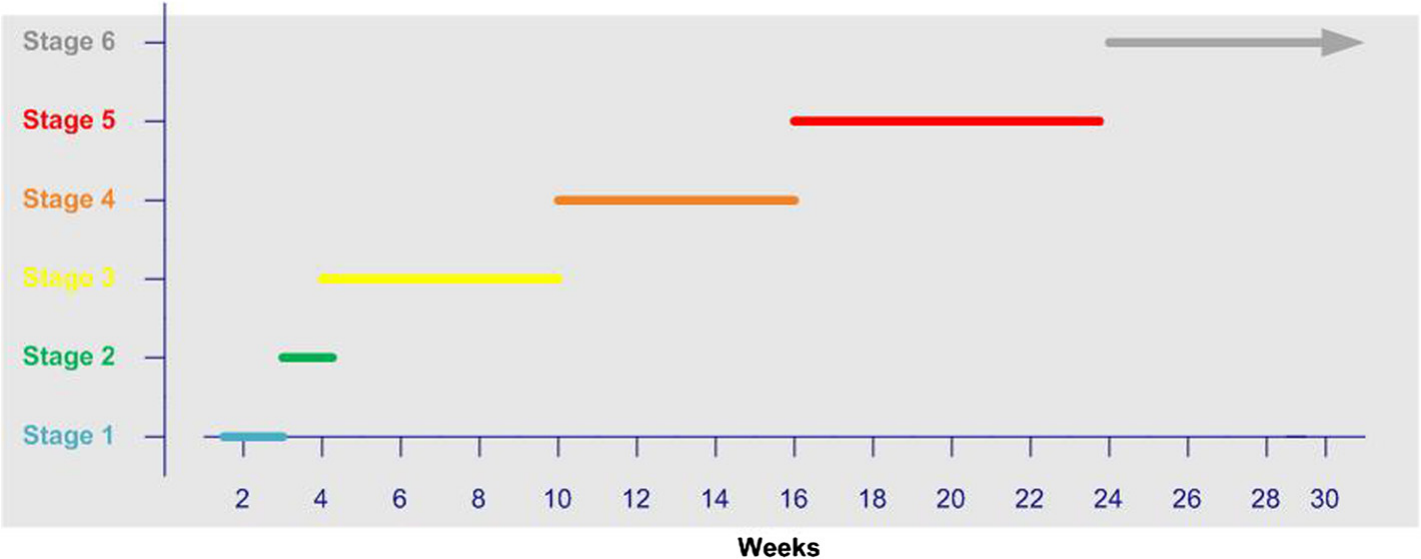
Weeks spent by the heart failure researcher in each stage of sense making.

In the case study, calculations of weeks spent in each sense making stage (see Figure 2) show that immersion in visualization tools (Stages 1 and 2) constituted only 13 percent of the total weeks of end-to-end analysis. In this 13 percent, the researcher in the case used two or more visual analytics tools at once during both Stages 1 and 2 in addition to external digital information.

In Stage 1 - characterizing genes conceptually – the three week total involved only 5.5 hours of real-time visual analytics. Of these 5.5 hours just 11 percent was spent studying the data to make sense of patterns and sub-groups. For the rest, the researcher divided the hours as follows: 62 percent laying out networks, 15 percent setting up and arranging views, nine percent formatting the copy-and-pasted tabular data in Word, and three percent color coding. The remainder of the weeks involved studying printouts of attribute tables of genes copied from the tools.

The other visual analytics stage – Stage 2, contextualizing related genes – consumed almost twice as many hours of hands-on visual analytics (9.3 hours) but just a week of overall time.

The calculations show that for 87 percent of the sense making flow the researcher was involved in the other four stages. Notably, it was in these other stages that she did the lion’s share of explanatory reasoning, with occasional returns to the visualization programs and views that had helped prepare her for this reasoning.

### Descriptions of sense making stages

**Figure 3:**
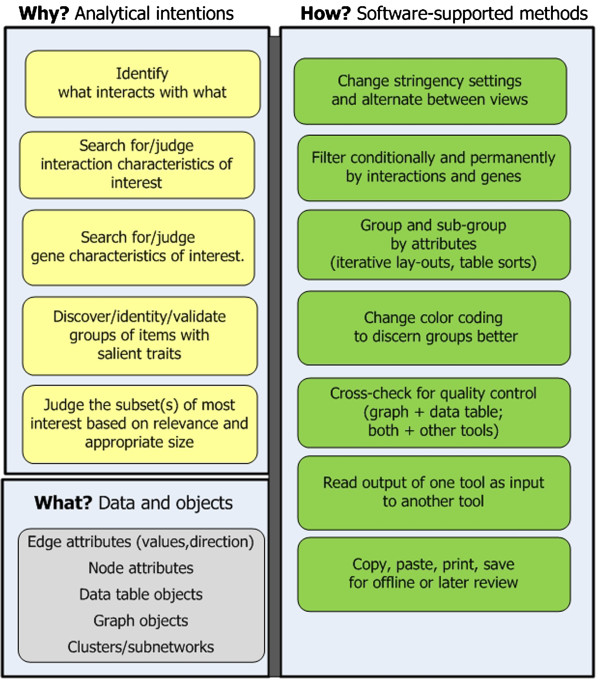
**Stage 1: Characterize genes conceptually.** The goal is to reduce expression genes to a meaningful subset.

**Stage 1: Characterize genes conceptually.** The analytical goal of this stage is to reduce the initial expression genes to a subset based on functional attributes relevant to the research problem (Figure 3). Approaches include:

– *Identify interactions between gene products.* Researchers query a protein-protein interaction (PPI) networks tool on a list of pre-processed (e.g. statistically significant) expression genes. The tool displays interactors and annotates genes (nodes) and interactions (edges) by attributes and/or derived values. Researchers read the graph and learn what gene products interact directly and indirectly [15].

– *Forage for information on conceptual traits of nodes and edges.* Researchers use a tool’s dynamically linked data tables and network to see the annotations of nodes and edges [16]. They select and focus on relevant, salient interactors. To reduce cognitive load, they capture, print, and manually annotate screen shots. For later offline analysis, they may copy, paste, and print tables into other programs (e.g. Word). They format and re-format copied data, as needed.

– *Filter on edge attributes*: Networks often can be reduced dramatically by filtering to only select edge attributes, e.g. sources citing interactions (e.g. pathway and disease databases), evidentiary strength (e.g. cited by three or more articles) or directionality. The graph then becomes more tractable [13].

– *Group, sub-group, and re-group nodes by function and filter by function, as needed:* Part of foraging for relevant attributes involves sorting tables and arranging networks into similarly annotated subnetworks. Much experimenting ensues to determine the most relevant groupings and to get clean borders. Many changes in layout occur [16]. Researchers save different network groupings for later review.

– *Overcome obstacles to achieving the right sized subset for further inquiry*. If a subnetwork of interest (e.g. a metabolism group) is highly connected it is difficult to subdivide further. To reduce highly connected networks researchers may read the member genes into another more stringently curated program. The high stringency reduces the number of interactions. Conversely, for sparse networks researchers may expand nodes to nearest neighbors and repeat “Characterize” methods on results [15].

**Stage 2: Contextualize related genes in biological events.** Using the subset selected in the previous stage the goal is to uncover broad outlines for a few biological stories involving some of the interactors in the subset. Researchers examine pairs of interactors , one pair at a time, in pathway contexts and biological events. This stage also has the goal of retrieving relevant citations during these contextual explorations. Citations collected now expedite literature searches later in subsequent stages.

**Figure 4:**
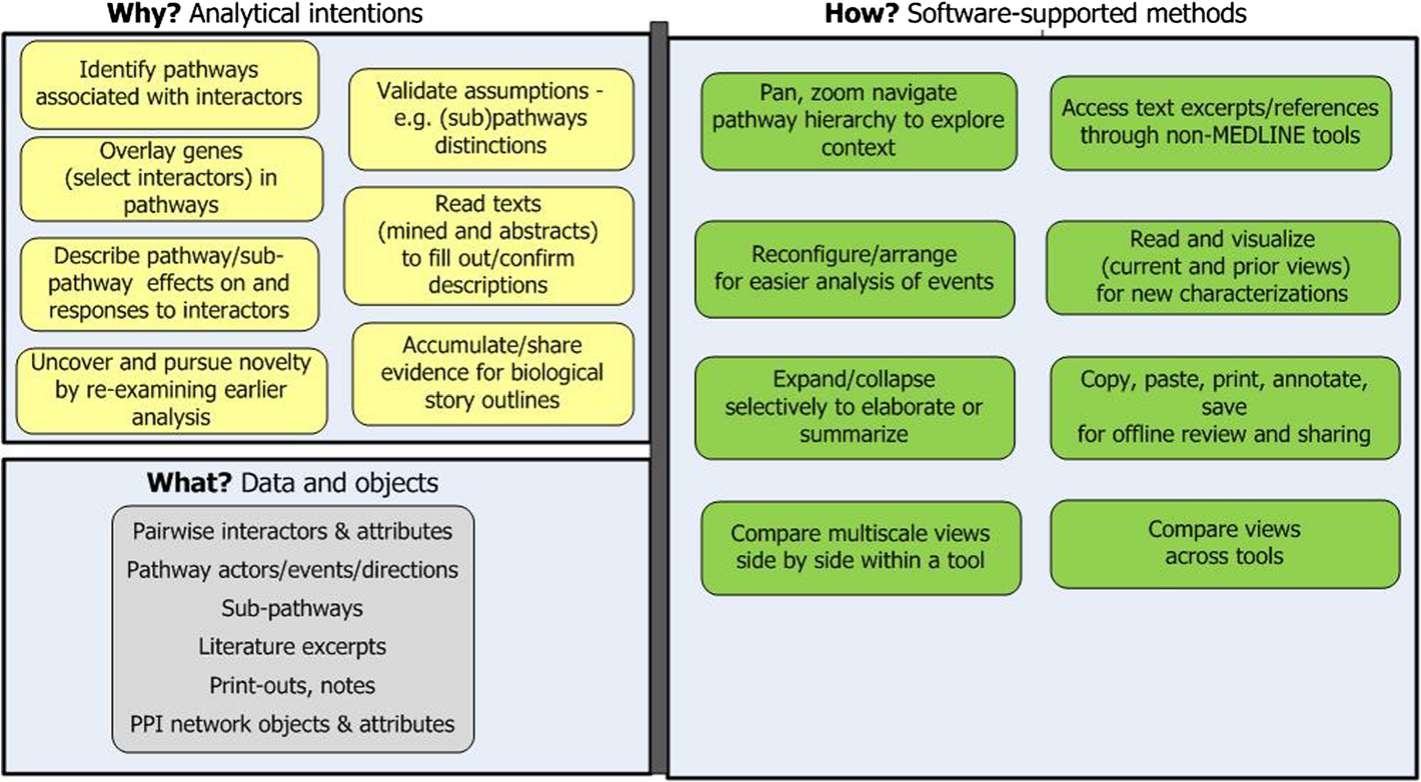
**Stage 2: Contextualize related genes in biological events.** The goal is to create story outlines.

This stage is more complex than “Characterize.” It involves close analysis of high dimensional, multi-scale relationships coupled with ongoing assessments of their relevance, potential novelty, cumulative implications, and credibility (see Figure 4). Approaches include:

– *Identify pathways in which interactors function.* Researchers query an interactive pathway program on one or two genes. It displays associated pathway (s) and sub-pathway (s), with the query gene (s) overlaid and highlighted on them [18].

– *Examine the role each pairwise interaction plays in context.* Researchers examine interactors at multiple scales and relate them to up and down stream regulatory events, other molecular pathway members, post-translational modifications, and directional (temporal) interactions and outcomes.

– *Validate the distinctiveness of sub-pathways and information they carry on interactors’ roles.* Researchers need to understand and validate how the sub-pathways they explore relate to each other, what differs between them, and what criteria a tool uses to sub-divide pathways. To do so, they access external information and place views of sub-pathways and parent pathways side-by side to compare them. Additionally, they refer back to the networks and earlier notes from the “Characterize” stage (Stage 1) to corroborate the interacting molecules with pathway views.

– *Manage perceptual orientation and trains of thought.* Maintaining focus and coherent thought is taxing when examining high dimensional data and multiple scales [17]. If researchers get disoriented they may abandon a program and seek workarounds (e.g. reading texts instead of visual analytics).

– *Forage textual information for interactors that suggest an interesting biological story***.** To put together “pathway stories,” researchers opportunistically shift between textual and visual modes. Ideally, they move seamlessly between visualization tools, PubMed abstracts, NLP annotations, and full texts. They delve into domain content, examining, for example, how normal and abnormal events differ, how transcriptional events affect or respond to translational modifications, and what trigger conditions occur.

– *Pursue promising unexpected leads by cross-referencing different or earlier tools*. Researchers welcome unexpected and potentially interesting findings. They often examine their implications by iterating back to prior moves within the current or prior stage [14].

– *Manage evolving knowledge and ideas by externalizing them in notes and diagrams.* To keep track of insights about possible story outlines as they evolve researchers take notes. They may annotate network print-outs, copy and paste excerpts from abstracts, other texts, and citations into laboratory notebooks; and enter additional notes in laboratory notebooks. They format copied data, as needed.

**Figure 5:**
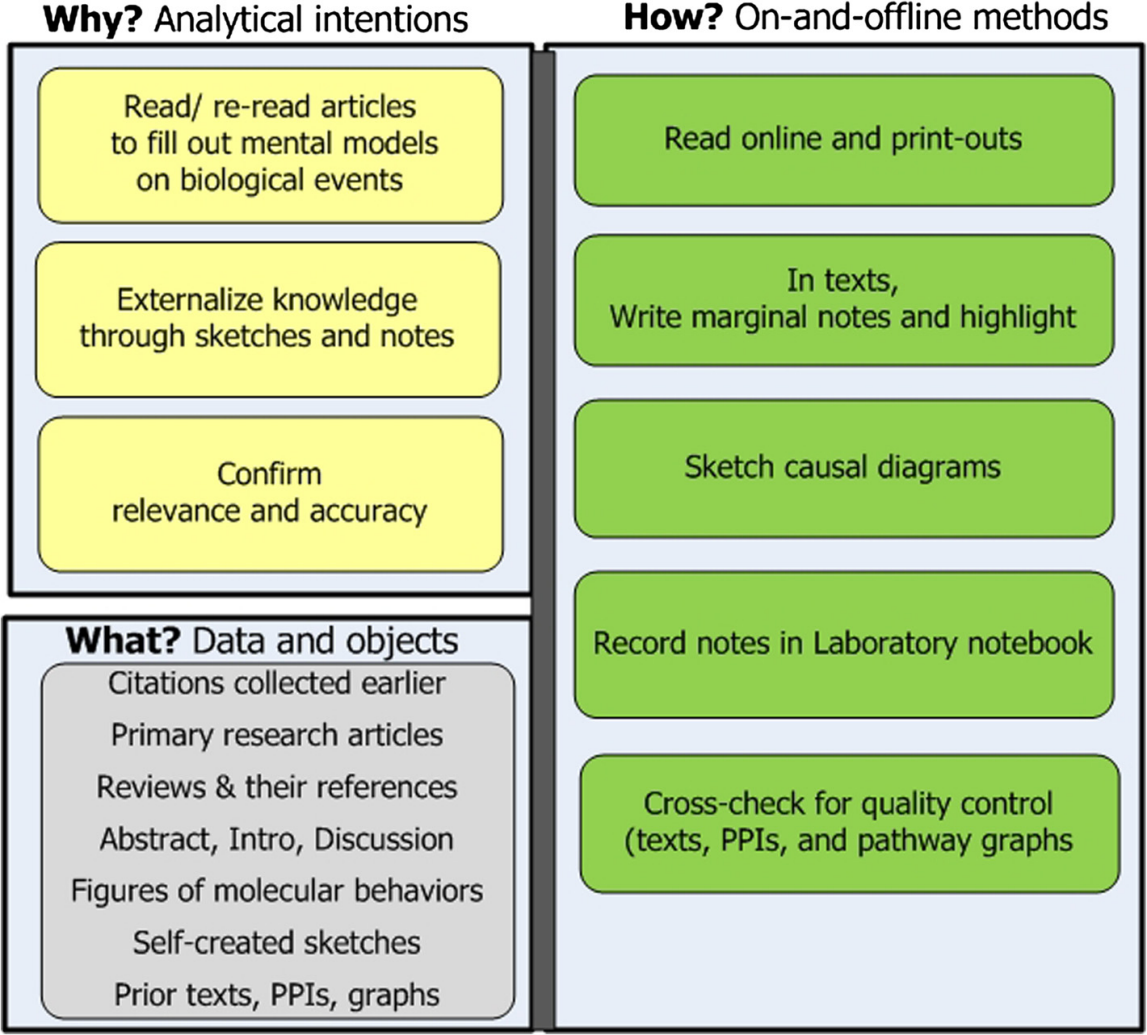
**Stage 3: Read for background.** The goal is to prepare for in-depth explanatory analysis.

**Stage 3 Read for background understanding and biological stories.** Reading in earnest now begins The goal is to internalize background knowledge as mental models. These mental models are necessary for subsequently constructing convincing narratives (see Figure 5). Approaches include:

– *Read articles from already collected citations and search for more.* Researchers read primary research articles and reviews. They read across sub-specialties, as needed (e.g. biochemistry, molecular biology, and bio-gerontology); and re-read articles several times.

– *Read portions of articles selectively***.** Reading focuses primarily on the article Abstract, Introduction, Discussion, and figures. Information of interest ranges from basic facts, such as gene synonyms, to complex relationships, such as interdependencies and modifications among functional relations. Figures illustrating the roles genes of interest play in these complex relationships are especially useful.

– *Externalize in-progress knowledge to assure understanding and to communicate it to others.* Researchers externalize their evolving knowledge to untangle and clarify the dense and multi-faceted relationships cumulatively obtained from the literature [21]. The externalizations may involve sketching causal maps - a convention in biomedicine for diagrammatically connecting numerous molecular actors through inhibitory and activating actions and outcomes [22-25]. Externalizing also includes highlighting texts, writing marginal notes, and entering questions and comments in laboratory notebooks. Diagrams and notes help in discussions with collaborators.

**Figure 6:**
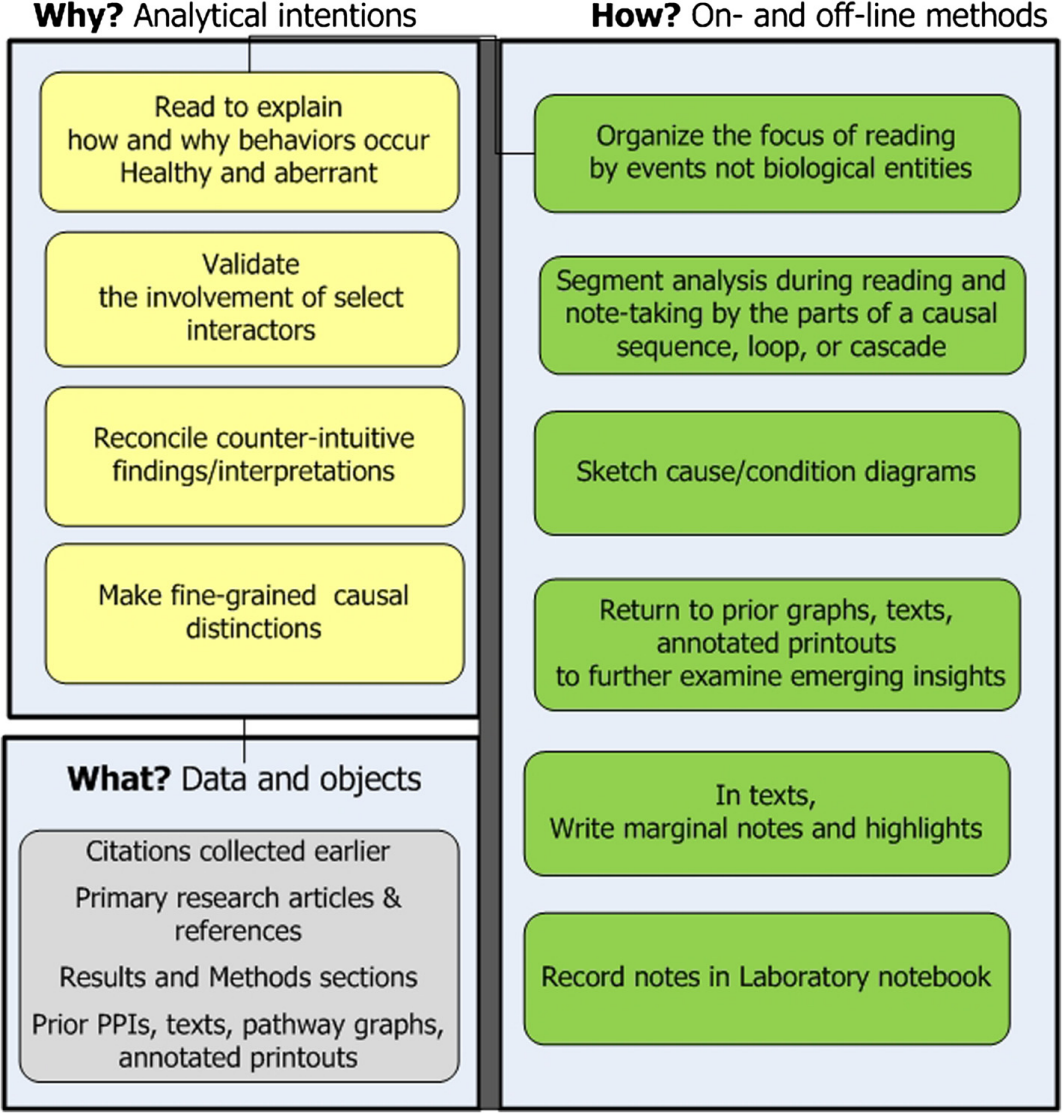
**Stage 4: Read to explain.** The goal is to construct rich narratives involving expression genes.

**Stage 4. Read to explain causes/conditions.** Stage 4 has the transformative goal of synthesizing cumulative research and knowledge into a causal and conditional biological story – a story from which to hypothesize later [13]. Reading focuses on how and why questions (see Figure 6). Approaches include:

– *Create new knowledge at a detailed level.* Reading mainly examines Methods and Results sections of primary research articles. Researchers follow chains of citations in articles of most interest. Issues of importance involve nuances of change or slight distinctions, e.g., varying effects that minor changes in durations and protein levels have on pathway events and modifications; or causal differences between distinct phenotypes of a disease.

– *Forage information for elaborations, confirmations, or validations.* Frequent references back to earlier PPI and pathway networks occur to help situate potentially interesting interactors in contexts of other genes and pathways and to validate that emerging insights and inferences are credible [14]. Cognitive dissonance likely occurs due to counterintuitive biological events (e.g. modulations causing a gene to perform opposing functions); and researchers strive to reconcile the tensions.

– *Externalize knowledge to relate relationships.* In addition to continuing with the same externalizing modes as earlier researchers now also deeply study their sketches (e.g. causal maps). They strive to understand how behaviors captured in different diagrams happen and how they connect to one another. Researchers return to prior articles and network views. They record open research questions into their laboratory notebooks.

– *Cumulatively build a biological story.* Researchers read for and build one story at a time. They stick with one if it seems plausible, novel and credible. Otherwise they move on to another possibility.

**Figure 7:**
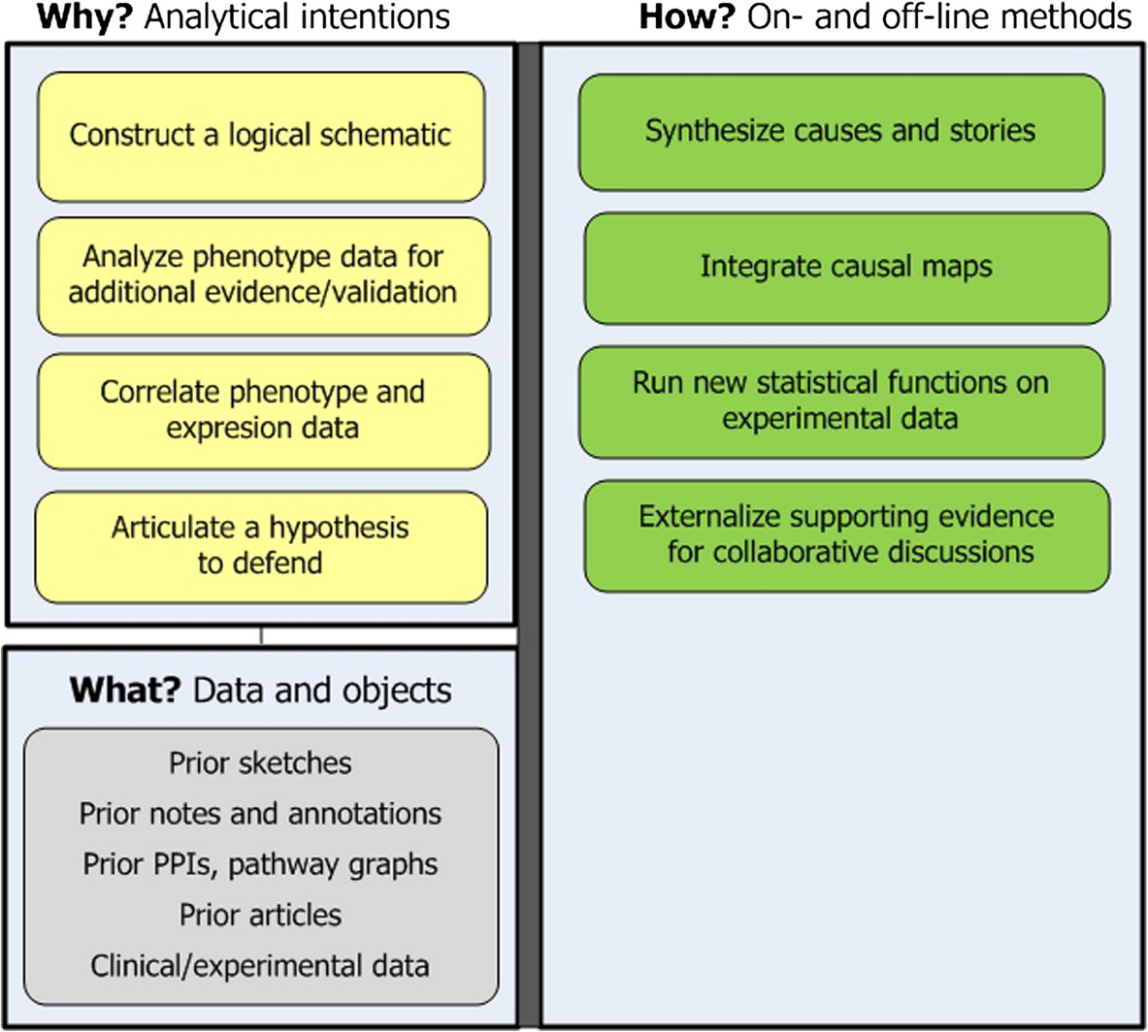
**Stage 5: Schematize and hypothesize.** The goal is to turn narratives into schematics into a hypothesis.

**Stage 5: Schematize and hypothesize.** Researchers construct logical schematics to turn rich narratives into explanations [21,25]. From them they articulate a hypothesis (see Figure 7). Approaches include:

– *Externalize knowledge as schematics.* Schematics consolidate and refine the novel biological story that researchers have been composing analytically. Visually, schematics may comprise a set of interrelated causal maps, each depicting part of the story. Schematics are cleaner than earlier hand-sketched causal maps, and researchers may craft them with drawing programs. The diagrams show functional interdependencies, conditions, and causal mechanisms and outcomes, all leading to an explanation of why disease-related processes occurred in the experimental samples.

– *Fill in evidential gaps by conducting new analyses and relating new and prior findings.* Schematics reveal weaknesses in the biological story, and researchers seek stronger evidence – often from the clinical data. Researchers may statistically analyze clinical/ phenotype traits and then correlate outcomes with certain molecular interactions to strengthen the hypothesis.

**Figure 8:**
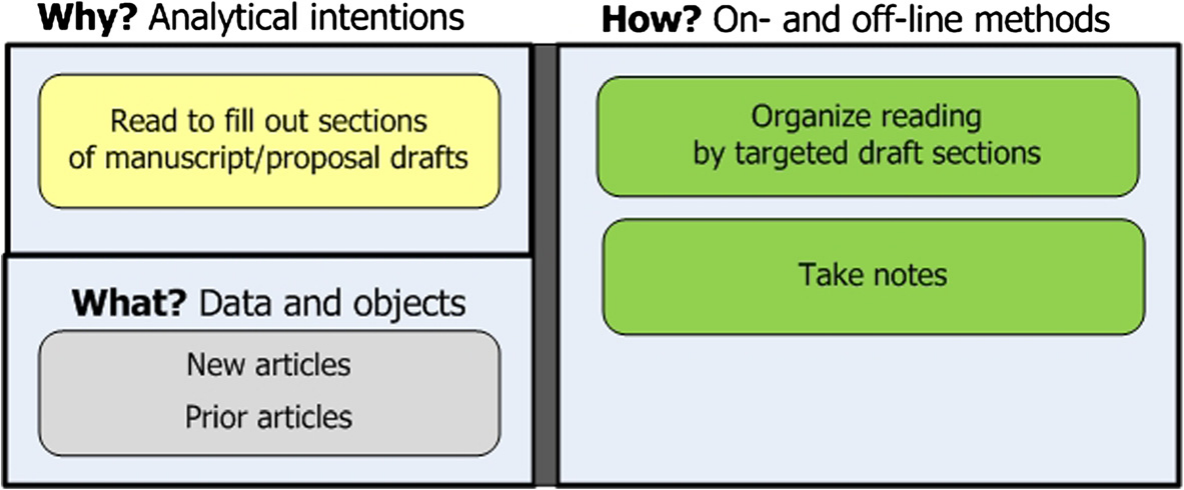
**Stage 6: Read to compose write-ups.** The goal is to substantiate manuscript/proposal drafts.

**Stage 6: Read and re-read to compose write-ups of findings**. The goal is to find just the information needed to write up findings in targeted sections of a manuscript of proposal (see Figure 8). Approaches include:

– *Forage for and understand information relevant to specific sections of drafts.* Researchers look at prior notes, readings, and new articles to substantiate the points they want to make in specific sections of an in-progress manuscript or proposal draft. They take notes while reading, and organize them by the sections of their draft.

### Guidelines for tool design

Our sense making model of expression-driven, -omics hypothesizing can deepen the bioinformatics community’s understanding of this problem space and the tool support scientists need for it. Case study findings suggest three issues to frame user-centered approaches to tool design. The first is that tools need to support scientists in iterating and unifying their moves across stages and not just within discrete portions of analysis. Iterations and recursions occur in the following categories of analytical activity:

• Query, retrieval, and filtering

• Visual analytics

• Externalized knowledge

• Primary literature

• Knowledge management.

The eight guidelines we propose below address these activities, as we indicate parenthetically below.

The second framing issue is that visual analytics is likely not yet scientists’ main means for constructing complex explanations in this class of analysis. Visul analytics holds the promise of augmenting explanatory reasoning [1]; but tools in bioinformatics have not yet delivered on this promise. Ideally, user-centered improvements to bioinformatics visualization tools will enhance the contextualizing needed for explanatory reasoning and to the integration of visualizations with important articles and other textual information for hypothesizing.

The last framing issue is that scientists use and want to use multiple tools. They do so to validate, shift perspective, and make and evaluate comparisons. Tools have to support this valued multiplicity and the continuous transfer of data it often requires. Framed by the three issues, specific guidelines for tool design based on our case include the following:

1. **Provide efficient filter and expand operations (Query, retrieve, and filter)**: Filter operations are essential to reducing data to a subset for further analysis, e.g. filtering by stringency, by attributes of individual genes, or attributes of subsets. Operations should allow filtering on orthogonal categories (not just terms/keywords) and on a variety of stringency parameters. Expansion operations are also essential, especially when networks are sparse. Operations should allow selective expansion and collapse. Effective and efficient filtering comes into play in the literature reading stages, as well, but in different ways, as detailed below.

2. **Provide interoperable data exchange (Query, retrieve, and filter)**: Programs should make it easy for scientists to move between tools (i.e. using output from one as input to another). Blurring the line between input and output helps achieve a smoother flow of analysis. Correspondingly, functionality to export data in a standard format is crucial; especially considering that in “Characterize” the heart failure scientist spent almost as much online time formatting as thinking.

3. **Support automated but still flexible network layout operations (Visual analytics)**: Manual network layouts can be extraordinarily time-consuming. Yet scientists’ abilities to construct appropriate mental models and to judge relevance and novelty depend on meaningful and accurate layouts. Tool operations should allow users to specify one or more traits for grouping and get layouts of like grouped with like.

4. **Support the analysis of different relationships in multivariate and multi-scale data (Visual analytics)**: Tools should afford multivariate what, why, and how inquiries; and multi-scale investigations should not spatially disorient scientific users. Overlays of genes on pathways are supported by various tools but few offer adequate support for making distinctions between sub-pathways and molecular roles within them. Also, transitions in displays across pathway hierarchical levels, for example, must be paced to coordinate with scientists’ mental transitions in changing levels. Tools also need to give scientists control over adjusting size and scale for both focus and context views.

5. **Provide multiple perspectives on the data, especially to foster comparisons (Visual analytics)**: To facilitate mapping across views, tool functionality should enable scientists to mark features or anchors across tools that are important to the research problem. It should also let them synchronize manipulations across views. Ideally, there should be more links and dynamic selection available across tools, perhaps with tools sharing a consistent knowledge base.

6. **Support the construction of external representations (Externalized knowledge)**: Many tools do not provide explicit support for the note-taking, diagramming, or annotating of visualizations that scientists value. Visual analytics tools should provide this support, ideally in ways that let scientists access or query these “externalizations” throughout the ongoing investigation. Invariably, scientists will end up taking notes and crafting sketches in many places. Designs for unifying such traces require more knowledge than currently exists about scientists’ patterns of note-taking and mark-ups for different purposes and representations they commonly use for various types of relationships.

7. **Support finding and annotating relevant documents (Primary literature)**: Some visualization tools give access to MEDLINE abstracts, NLP, and citations but rarely do they let scientists see and group potentially relevant texts and/or figures by conceptual relationships. Such functionality would be useful. Also, because it is likely that researchers know the sections of articles they want for a particular stage and purpose of reading, it would be expedient to let them select extracts from desired sections when full text NLP is available.

8. **Record the investigation history (Iteration and knowledge management)**: Given the numerous times researchers return to earlier moves and tools for a variety of different purposes, tools need to support researchers in keeping track of the ongoing investigation. Tools should automate the recording of investigation history and let researchers add their own annotations and go back to previous states in an investigation. Additionally, the investigation history should be accessible in an intuitive and easy to use interface and not increase a researcher’s cognitive load.

### Limitations and future directions

The proposed guidelines specify necessary support for efficient, effective, coherent, and complete sense making. They do not endorse, however, any specific tools, suites of tools, features, or user interface designs as being better than others. Moreover, some aspects of sense making fall outside the scope of our research design but are nonetheless critical to sense making success. For example, collaborative interactions and collaborative activities to create externalized representations play important roles in clarifying and deepening researchers’ mental models and subsequent inquiry directions. Also outside our scope is an in-depth look at the distinct content structures and processes in reading as well as in-depth modes modes of reasoning involved in moving from description to biological narrative to explanation and hypothesis. We did not directly observe the synthesis and novel insights during Stages 3 and 4 that were critical to explanatory reasoning. Rather our scope only enabled us to uncover, for example, that narratives became prominent when the researcher in the case had to tease apart dynamic relationships and outcomes (beyond pairwise interactions) that were too complicated to hold in mind at once and understand (due, in part, to insufficient software support).

We believe that future research needs to continue to build out the stages of sense making and corresponding requirements for user-centered tools. It needs to tie visual analytics as well as semantically rich information from text mining to actual sense making. More empirically-based models of other workflows and more refinements and/or alternatives to our own are needed.

## Conclusions

Sense making models have proven useful for understanding analysts’ application-level tasks and for developing user-centered tools that can facilitate and enhance these tasks in an integrated way. We believe that the model we presented in this paper is an important first step to introduce sense making models to the bioinformatics community. We do not claim that our model accurately represents every researcher’s workflow for analyzing a set of genes and generating a hypothesis to explain disease mechanisms. Instead, we believe that our model accurately represents a subset of researchers’ workflows and can serve as the foundation for future refinements and developments of competing models. Our proposed model provides a “language” that researchers can now use to define tasks and workflows and to build tools that specifically support those identified tasks and workflows as a whole and not only in separation. Therefore, the contribution of our work is not only the sense making model itself but also the introduction of sense making models to the bioinformatics community.

## Methods

For six months we observed and interviewed an IRB-consented biomedical researcher as she worked with expression data to generate a plausible and credible hypothesis about why some non-ischemic heart failure patients failed to respond to beta-blocker treatment. She identified non-responders from her team’s clinical data and explored their richly annotated expression data using a number of bioinformatics and MEDLINE tools. She collaborated offline with her team of heart failure specialists. They generated a hypothesis at the end of the case study, wrote a grant proposal, and began a manuscript.

Using Camtasia screen capture software, we gathered 15 hours of video and audio data on the researcher’s interactions with visualization tools and her verbalized thoughts as she worked with the visualizations. We interviewed her once a month for more details about her inquiries and took copious notes. We also interviewed her several times at the end of the case study to validate interpretations and gather more details. (20 hours total of interviews). We collected copies of her laboratory notebook (55 single spaced pages, including screen captures) along with 40 pages of other hand-written notes and sketches, and the PubMed articles that she read and annotated. The researcher’s offline collaborations were outside the scope of our analysis.

To analyze the visual analytics data, we transcribed the Camtasia tapes and conducted several holistic readings of the transcriptions, interview notes, and relevant portions of the laboratory notebook. We then abstracted patterns and themes relevant to our sense making research goals and informed by relevant theories and frameworks on the cognitive and technological demands of complex problem solving with interactive visualizations [26]. We categorized the researcher’s goals, analytical tasks, sub-tasks, and actions; restructured them into a flow of sense making reasoning and behaviors; and identified the information used as the inputs and outputs of various analytical tasks. We tied information and actions to tools and tool features and framed task flows by analytical intentions. From time stamped data, we quantified time on tasks and sub-tasks.

To analyze the portions of the workflow that involved reading and making sense of PubMed articles we examined the laboratory notebook, annotated articles, hand written notes, and relevant interview responses. We examined 40 abstracts that she cited or copied in her notebook annotated with points of interest and read the corresponding articles for more detail on the issues of interest. We distinguished and characterized types of reading based on analytical intentions, modes of reasoning and note-taking; and focus of attention (i.e. article sections and content). We estimated time on reading-and-thinking based on laboratory notebook entry dates and the researcher’s self-reported durations during interviews. We integrated results from other relevant bioinformatics and visual analytics research with our findings and finalized a sense making model and design guidelines.

## Competing interests

The authors declare that they have no competing interests.

## Authors’ contributions

BM designed and carried out the field research, the data collection, and the data analysis. BM and CG collaborated on interpreting findings and determining how to organize and report results. They collaborated on developing, rendering, and refining the sense making model. CG described the implications for tool design. Both authors drafted the manuscript and read and approved the final version.

## Supplementary Material

Additional file 1 **Heart Failure Case Study: Narrative of Progressive Discovery.** A detailed narrative specific to the heart failure problem and insights is presented for each stage of sense making. To facilitate recall of each stage and cross-reference from the prose, a simplified rendition of the sense making model is included. Click here for file
