# Supplementary material for: Scientists’ sense making when hypothesizing about disease mechanisms from expression data and their needs for visualization support
Source: BMC Bioinformatics. 2014 Apr 26;15:117. doi: 10.1186/1471-2105-15-117 (PMC4021544; doi:10.1186/1471-2105-15-117)
Supplement: Additional file 1 — Heart Failure Case Study: Narrative of Progressive Discovery. A detailed narrative specific to the heart failure problem and insights is presented for each stage of sense making. To facilitate recall of each stage and cross-reference from the prose, a simplified rendition of the sense making model is included. [file 1471-2105-15-117-S1.pdf]

Additional Material

Heart Failure Case Study: Narrative of Progressive Discovery

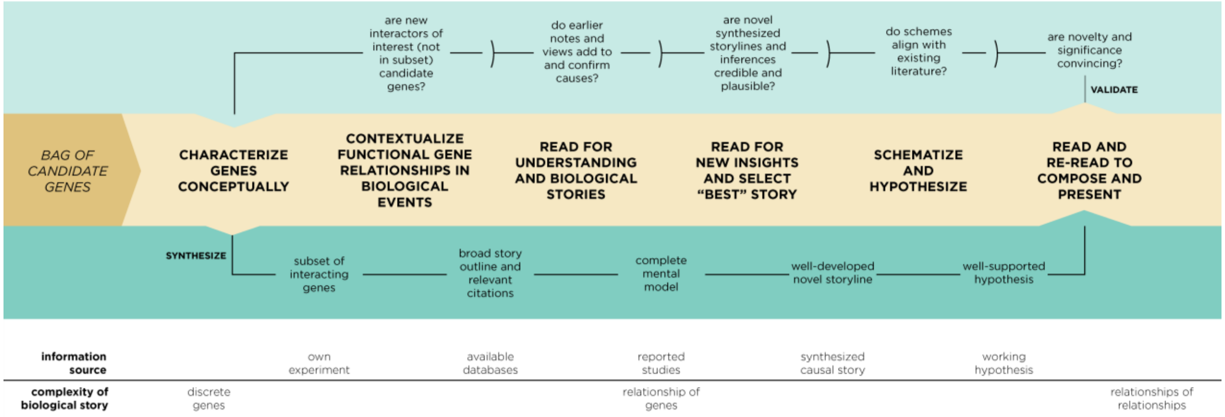

Figure 1. Simplified rendition of the sensemaking model for easy cross referencing to the narrative.

Stage 1. Characterize genes

The researcher started by examining 93 differentially expressed genes from heart failure patients who did not respond to beta blockers. She read them into a network program - a Cytoscape 2.8 plugin with its own integrated database. The program determined interactions among gene products and annotated both the interactions (edges) and genes (nodes) by attributes drawn from numerous databases and other metadata sources. The displayed network was large – 93 genes with 1011 interactions; and the researcher filtered it to only the interactions that were drawn from pathway and disease databases. The result was 44 genes with 745 interactions, which the researcher now separated into three clusters (see Figure 2) based on common cellular functions among interactors (metabolism, apoptosis, and "other"). As she worked, she filled in information that she needed by going to external sources, e.g. the Genetic Association Database (GAD) (geneticassociationdb.nih.org), the Pharmacogenomics Knowledge Base (PharmGKB) (pharmgkb.org), Online Mendelian Inheritance in Man

(OMIM) (ncbi.nlm.nih.gov/omim), NCBI Entrez Gene (ncbi.nlm.nih.org/gene), and Interpro (ebi.ac.uk/interpro).

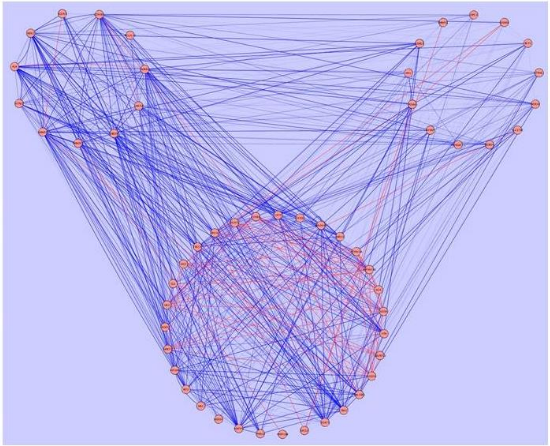

Figure 2. Three sub-networks grouped by shared attribute. The one in the forefront has 31 highly connected genes enriched for metabolism.

By far the largest share of the genes were enriched for metabolism (31/44 genes) but this group was highly interconnected. Therefore, she read the 31 genes into String, a stringently (manually) curated, web-based network tool (http://string-db.org/). Its results displayed only ten interacting genes - four pairwise interactions and two triplets. So far she had no coherent story or biological explanation to tie them together or to tie them to non-responsiveness to treatment in non-ischemic heart failure.

Stage 2.Contextualize select interactors

The researcher studied the ten interactors displayed in String in their respective pathways. She hoped to find if roles they played shed light on such questions as: Where does the heart draw energy from? How does it use fuel when it is in trouble? What roles do fuel-use processes and associated pathways play in beta blockade? Is there enough known and still an open question about heart metabolism to warrant continued inquiry into these genes?

She overlaid one pair of interactors at a time on annotated pathways and sub-pathways in Reactome Analylzer to examine them within and across biological scales (http://www.reactome.org). She struggled to keep oriented in the workspace. She interacted with several programs and external

sources of information, including PubMed and NLP-derived excerpts; thematic details from Wikipedia (e.g. chondogenesis of the heart); more node and edge annotations; and details from external sources about events recurrently associated with the interactors (e.g. hypoxia-related metabolism in general and hypoxia connected specifically to bacterial infections, muscle cells under stress, and circadian rhythms). She continuously externalized knowledge, for example, as annotations to network printouts, notes and questions in her laboratory notebook, and copied-and-pasted excerpts and screen shots.

The pathway program turned out to be too dense and confusing. She could not easily see context and details at once, could not readily compare visual displays of sub-pathways, and could not keep oriented while navigating across pathway interactions. Programmatic transitions and juxtapositions did not leave visual traces of starting points, which resulted in a heavy burden on the researcher's visual memory. Finding the burden too great, she abandoned the program in favor of using textual data drawn from String, the manually curated network visualization program she used earlier. She coupled text explorations with frequent cross-references between the network programs from earlier to visually situate the interactors she read about.

Her analysis uncovered that sustained hypoxic conditions (deprivation of adequate oxygen) triggered abnormal activation and inhibition loops between some of the interactors. The outcomes of these interactions initiated other transcriptional and post-translational modifications that could be tied to maladaptive processes in the myocardium for heart remodeling. A breakthrough occurred when she realized that a gene missing from her original list of expression genes was instrumental in these processes. She noted:

> The one node HIF1A [hypoxia-reducible factor 1 alpha) and the hypoxia pathway might be a breakthrough. They might be pulling together themes that we're seeing in responders and non-responders. HIF1a hasn't been differentially expressed but is regulated at the proteomic level [so wouldn't be in our gene list]. Upstream genes competitively bind with the protein. When the upstream genes are down-regulated the protein is free to do translation downstream. How it gets modified varies – and it's that that I have to look into.

With this new information about HIF1A, the researcher returned to the network programs used in the Characterize stage. In the stringently curated program she expanded the ten interactors to their nearest neighbors and got a display of several sub-networks rather than the sparse pairs and triplets. In it she found three genes mentioned frequently in textual information that she had skim-read during her contextualizing analysis. In this nearest-neighbor network, the researcher now saw more pairwise interactors to contextualize and continued doing so thoroughly with each pair. She printed the nearest-neighbor network and annotated it as she worked. She ended this stage with several plausible story outlines and 30 citations to articles. The story outlines variously plotted how sustained hypoxic conditions activated different processes, all of which could adversely affect adaptive processes in the myocardium required for heart remodeling. Non-responsiveness to beta blockade fit in because beta blockers – when they worked - stopped or prevented maladaptive heart remodeling. As the researcher noted, “A lot of things are running through the same hypoxia pathway. But there are a number of ways in which things can be turned on and off – age, stress, downstream effects, muscle cells under stress, and circadian rhythms. I have to follow up on them.” At the end of this stage the researcher’s annotated printout of the nearest neighbor network showed which interacting expression genes potentially were associated with ways in which the heart was adversely affected by calcification, chondrogenesis, circadian rhythm, lipidogenesis, angiongenesis, Alzheimer’s disease, hypoxia-induced hypertension, and fibrosis and fat tissue in their muscles.

Stage 3. Read for background knowledge

At this point the researcher put the development of specific storylines on hold. She first had to deepen her background understanding. She printed and read 16 of the 30 articles that she had gathered earlier as citations or abstracts. Broadly and largely aided by review articles, she filled in her mental models of inhibitions to HIF1A’s “normal” degradation and the streams of effects this inhibition had. At a more detailed level and largely from primary research articles, she came to better understand

ischemic preconditioning, adverse effects of bacterial infections or aging heart phenotypes, and effects of inhibited adipogenesis on fatty acid metabolism. She read each article first for its gist and then again with the goal of “putting information in my brain.” She externalized her progressively deepening knowledge in many ways. Notably, she sketched a total of 11 causal maps - a sketch for almost every article - to capture relationships of relationships involving the expression genes (See Figure 2 below). A figure in one of the articles was a turning point. It enabled her to extensively fill out her mental model of events and effects.

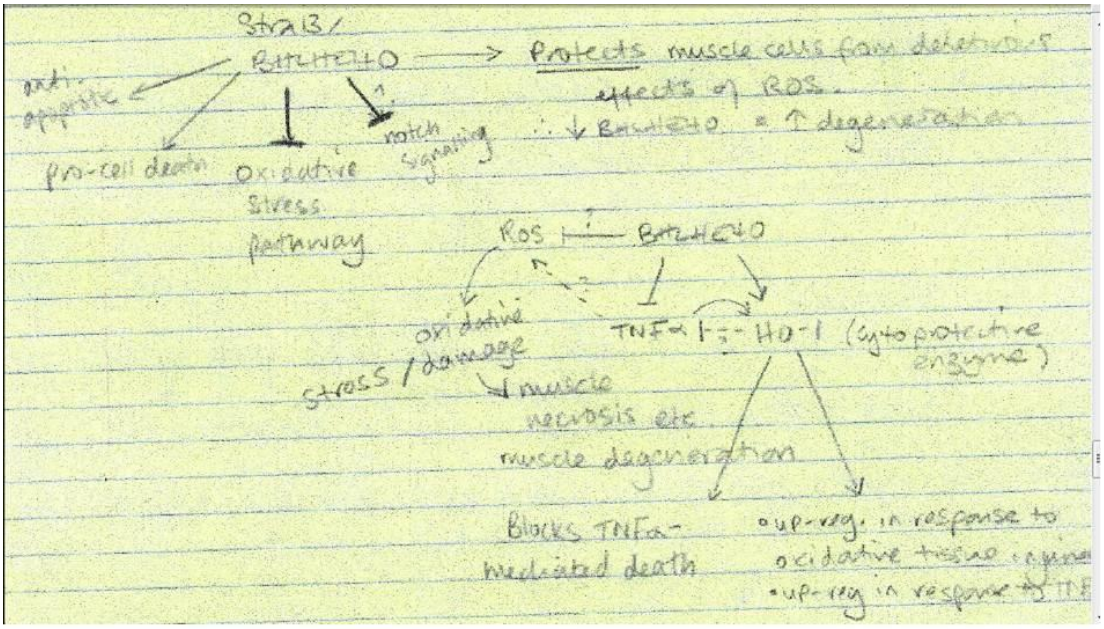

Figure 2. Causal map sketches drawn from the following text: The molecular mechanisms by which Stra13 regulates oxidative stress remain to be investigated. Stra13 is expressed at low basal levels in myoblasts, and its overexpression is sufficient to down regulate TNFa expression and up-regulate HO-1 expression. In response to oxidative stress, it is possible that Stra13 expression/activity is modulated, that allow it to maintain reduced TNFa levels, as well as increase expression of cytoprotective enzymes such as HO-1. Together these results suggest that Stra13 regulates muscle integrity by providing critical protection to muscle cells from oxidative damage…Post translational modifications or interaction with different protein partners may modulate Stra13 and enable it to perform seemingly opposing functions” (Vercherat et al, 2009, 4314)

Stage 4. Read to explain causes/conditions

Having assimilated ample background knowledge, the researcher now turned to the literature to ask: Why did the behaviors captured in the causal maps occur? How did the processes and events captured in maps connect to one another? Why and when was it reasonable to assume that expression

genes played active roles in causal sequences? What did hypoxia-related events have to do with beta blocker responsiveness and how, when and why was responsiveness affected?

She read 15 research articles, two of which she retrieved by following article citations. She continued sketching causal maps as she read. Now, however she distinguished between causal profiles of hypoxia and ischemia and causal forces leading to failed instead of recuperative heart remodeling. She also read research studies that helped her differentiate various heart phenotypes (e.g. hibernation vs infarction) relevant to the expression genes from the non-ischemic cardiomyopathy patients. She dug into Methods and Results sections to find, for example, if criteria that a study used to identify patients with ischemic cardiomyopathy could be useful to her own analysis. She also frequently returned to earlier tools and printouts to see how genes were positioned in relation to each other. She found that she should not pursue story lines associated with bacterial infections, tumors, and adipogenesis because the related expression genes were too many hops away from HIF1A in the PPI networks.

She progressively came to a novel story tied to the hypoxia pathway. Due to its overlap with a number of other pathways, it was likely implicated in these patients and not only in more commonly hypothesized ischemic cardiomyopathy patients. She noted:

> Metabolism, death, aging, apoptosis, and switching between sugar and fat are important in failing hearts. For example, if the switch is one way it leads to remodeling and it makes a person get sicker. HIF1a can be triggered not just because the heart is struggling but also by the remodeling process or by high pressure, high force, or muscular stress. Oxygen depletion plays a role, and beta blockers help. Maybe the hypoxia pathway has been seen in different patients under different scenarios before. But the pathway may be implicated in a broader collection of patients that those that are just hypoxic because of these other triggering processes. It's not a massive leap. There's some prior evidence. But there hasn't been explicit evidence or how it all might be working.

Stage 5. Schematize and hypothesize

Ready now to generate a hypothesis from her analysis, synthesis, and inferences, the researcher abstracted causes and outcomes into logic representations , which we refer to as schematics

(See Figure 4). She also ran more statistical analyses on the patient data to see if results supported the story and hypothesis she was putting together. Specifically, she wanted to see if certain phenotypes correlated with genes in the causal schematics that seemed most influential – i.e., BHLHE40, HIF1A, EGNL3, ADRB2, VEGF (an angiogenic growth factor), and p53.

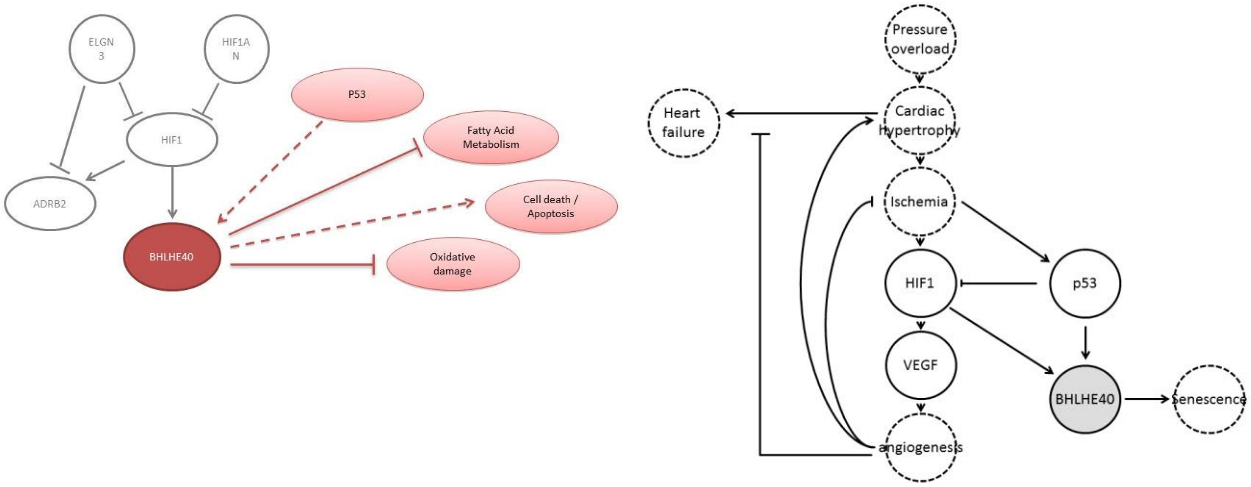

Figure 4. Two schematics with different levels of detail.

Heatmaps of correlations provided compelling evidence in another watershed moment in this analysis. Within non-responders, the heatmaps clustered into four groups, and one was striking. This cluster of patients had the lowest rates of left ventricle ejection fraction responses - indicating less likelihood of improving with beta blockade - and the widest QRS intervals in electrocardiograms. They also had significantly higher expression of HIF1A, and lower expression of VEGF, BHLHE40 and P53 relative to HIF1A. This group became the final limiting case for hypothesizing. The hypothesis basically gave details about up and down stream of molecular events in cells that led to disruptions in pathway signaling, which was traceable to pathological phenotype traits in patients who did not respond to treatment. All the experimental genes singled out in the patient correlation analyses participated in some way in "uncoupling molecular signaling in myocardial response to hypoxia associated with widened QRS interval and reducing likelihood of LVEF improvement with beta blockers." The exploratory analysis had produced novel claims about a network of interacting genes influencing particular phenotypes of non-ischemic heart failure.

Stage 6. Read and re-read to compose write-ups of findings

The research team had a strong enough hypothesis and design for research to test it to The next try to get funding and draft a manuscript on findings. The researcher returned to notes and articles that she had read earlier and some new ones to substantiate the various sections of the write-ups.
